# KlimaNot—Effects of Climate Change on Emergency and Acute Care: Protocol for a Multicenter, Registry-Based Observational Cohort Study

**DOI:** 10.2196/82267

**Published:** 2026-06-25

**Authors:** Kai Heimrath, Mirjam Rupprecht, Madlen Schranz, Timon Klein, Jenny Unterkofler, Tobias Schilling, Jonas Bienzeisler, Miriam Hertwig, Johannah Cramer, Merle Potzauf, Thea Laurentius, Wiebke Schirrmeister, Cornelius Bollheimer, Sebastian Sager, Rainer Röhrig, Felix Walcher

**Affiliations:** 1 Institute for Public Health in Acute Medicine Otto-von-Guericke University Magdeburg, Saxony-Anhalt Germany; 2 Department for Infectious Disease Epidemiology Robert Koch Institute Berlin Germany; 3 Emergency and Acute Medicine Charité – University Medicine Berlin Germany; 4 Institute of Public Health Charité - University Medicine Berlin Germany; 5 Institute for Mathematical Optimization Otto-von-Guericke University Magdeburg Germany; 6 Clinic for Anesthesiology RWTH Aachen University Aachen Germany; 7 Department of Interdisciplinary Acute, Emergency, and Intensive Medicine Klinikum Stuttgart Stuttgart Germany; 8 Institute for Medical Informatics RWTH Aachen University Aachen Germany; 9 Center for Clinical Acute and Emergency Medicine RWTH Aachen University Aachen Germany; 10 Regional Climate Office German Meteorological Service Potsdam Germany; 11 University Clinic for Geriatrics Klinikum Oldenburg Oldenburg Germany; 12 Clinic for Geriatrics RWTH Aachen University Aachen Germany

**Keywords:** climate change, routine health data, emergency department, heat, syndromic surveillance

## Abstract

**Background:**

Due to climate change, the population and health care systems face an increasing burden of weather-related health risks. Emergency departments (EDs) are one of the first points of contact for acute and emergency care and insights into population health. Previous research has demonstrated that climate change-based weather phenomena have an impact on ED usage and morbidity. However, research shows inconsistent results for some weather phenomena and disease groups, and no corresponding evidence is yet available for Germany.

**Objective:**

This study aims to investigate the association between climate-related weather conditions and ED usage and morbidity in Germany. It focuses on identifying particularly vulnerable patient groups, developing indicators for syndromic surveillance, and testing prediction models to support clinical and public health decision-making.

**Methods:**

KlimaNot is a multicenter, registry-based observational cohort study with retrospective and prospective components. The primary analysis is a prespecified retrospective evaluation using routinely collected encounter data from the German National Emergency Department Data Registry (AKTIN), comprising approximately 6.35 million ED visits from up to 56 EDs (2019-2024). Hospital-level environmental exposures (eg, temperature and selected air pollutants) will be linked to each participating ED based on location and summarized to daily metrics. The primary endpoint is daily all-cause ED visit volume at the hospital-day level. Secondary endpoints include hospital admission probability, syndromic and diagnostic case-mix, referral source and mode of transport, and routinely recorded proxies of clinical severity (eg, triage acuity). Heat effects will be quantified using models allowing for nonlinear exposure–response relationships and delayed (lagged) effects, with adjustment for site, seasonality and time trends, weekday, and public holidays; effect modification by age, sex, multimorbidity proxies, and area-level socioeconomic deprivation will be assessed. Additional analyses include a predefined case study for the region Stuttgart, leveraging extended longitudinal and more detailed pathway data, development and validation of heat-sensitive syndromic surveillance indicators, evaluation of short-term forecasting models of ED usage, and an ancillary prospective geriatric substudy collecting patient-reported and functional outcomes to better characterize vulnerability in the oldest-old.

**Results:**

Retrospective data analyses are ongoing and scheduled for completion by April 1, 2026. The prospective study will expect results by September 1, 2026.

**Conclusions:**

This study will provide the first robust evidence on the impact of climate change–related weather conditions on ED usage and morbidity in Germany. The findings aim to support early detection, preparedness, and targeted protection strategies for vulnerable populations and inform clinical and public health decision-making.

**Trial Registration:**

German Clinical Trials Registry DRKS00033214; https://drks.de/search/en/trial/DRKS00033214 and German Clinical Trials Registry DRKS00037822; https://drks.de/search/en/trial/DRKS00037822

**International Registered Report Identifier (IRRID):**

RR1-10.2196/82267

## Introduction

### Background

As a result of climate change, there is an expansion of weather-related health risks affecting the population and challenging health system actors. Heat waves can lead to increased mortality [1,2]. The unusually high summer temperatures in Germany between 2018 and 2020 resulted in a significant number of deaths, with approximately 8700 heat-related deaths in 2018, 6900 in 2019, and 3700 in 2020 [2]. Regarding morbidity, it is already known that individuals afflicted with respiratory disorders, including chronic obstructive pulmonary disease and asthma, are susceptible to the adverse effects of high temperatures [3]. This susceptibility is further exacerbated by environmental pollutants that heighten sensitivity to inflammatory reactions [4,5]. Cardiovascular diseases such as myocardial infarction and decompensating heart failure [6-8], as well as acute kidney injury resulting from dehydration, are associated with high temperatures [9,10]. Furthermore, natural disasters and extreme weather events have been associated with psychological distress, cognitive impairment, and headaches [11,12].

Patients with multiple comorbidities, older people, children, and adolescents are particularly vulnerable during extreme heat [13-15]. Masselot et al [16] examined the impact of extreme temperatures on different age groups in 854 European cities. Their findings emphasize that heat-related health risks increase with age. Specifically, older adults (65 years and older) are more vulnerable to extreme temperatures, experiencing higher relative risks of mortality compared to younger adults. In the United States, it has been shown that extreme heat results in a higher occurrence of emergency department (ED) visits due to heat-related illness, kidney diseases, and mental disorders among both younger and older adults [17]. Bernstein et al [15] demonstrated that extreme heat during the warm season has led to a significant increase in ED visits at children’s hospitals in the United States. The analysis revealed that high temperatures are associated with a 17% increased risk of all-cause emergency visits. The associations were particularly pronounced for heat-related illnesses such as dehydration and electrolyte disorders (83% increased risk), bacterial enteritis (35% increased risk), and ear infections (30% increased risk). Children with comorbidities, genetic susceptibilities, or those who face adverse social determinants of health are particularly at risk [15]. Thus, the question of how climate change-related weather influences the emergency and acute medical care needs of different socioeconomic groups becomes increasingly important for health care research.

Although there is already extensive evidence on the impact of weather and the environment on population health, the influence of weather-related factors on the use of EDs is still not sufficiently understood**.** While there is unambiguous evidence of a positive association between high ambient temperatures and ED visits due to directly heat-sensitive diseases such as dehydration and electrolyte imbalances, there is inconclusive evidence on the relationship between temperature and cardiovascular, renal, and respiratory diseases [18-20]. EDs serve as a central element of the German health care system and provide crucial insights into current emergency and acute medical care trends. Given the ubiquitous effects of global climate change and the existing evidence, we hypothesize that climate-change-related weather conditions, such as extreme heat days, significantly influence population health and the usage of EDs in Germany. Moreover, we assume geographical differences and a group-specific impact on children, older, and patients with multiple comorbidities.

### Objectives

We will link routine ED encounter data from the German National Emergency Department Data Registry (AKTIN Registry) with high-resolution environmental data to investigate heat-related impacts on acute and emergency care in Germany. The study follows a hierarchical objective structure with a prespecified primary retrospective analysis, secondary clinical objectives, and additional exploratory components (syndromic surveillance and prediction modeling).

### Primary Objective (Prespecified Retrospective Analysis)

Objective 1 (O1): the primary objective is to quantify the association between ambient heat and acute-care demand in Germany, using a prespecified retrospective analysis of daily ED visit volumes as primary outcomes. We will compare heat days with comfort days, quantify exposure–response relationships, and characterize immediate and delayed effects of heat over predefined lag windows as the primary objective. In addition, we will identify temperature thresholds at which ED attendances and hospital admission probabilities increase and assess whether these associations differ across vulnerable patient groups, with a focus on children, older, and people with multiple comorbidities.

### Secondary Clinical Objectives (Retrospective; Secondary Endpoints)

Objective 2 (O2): we will analyze hospital admission rates related to heat exposure.

Objective 3 (O3): we will examine shifts in syndromic and diagnostic patterns on heat days.

Objective 4 (O4): we will assess pathways to emergency care, including referral source and mode of transport, by applying suitable models for compositional or proportion outcomes.

Objective 5 (O5): we will assess clinical severity at ED presentation using routinely recorded proxies, including triage acuity and high-acuity presentation rate.

### Ancillary Prospective Geriatric Substudy

Objective 6 (O6) (ancillary prospective substudy; separately registered): we will characterize patient-reported heat-related symptoms and functional impact in oldest-old patients (aged 85 years or older) to better describe vulnerability beyond routine registry variables.

### Exploratory Objectives (Hypothesis-Generating and Methodological Components)

Objective 7 (O7): we will assess the suitability of routine ED data for continuous syndromic surveillance of weather-sensitive morbidity. The initial focus is on the development of an indicator monitoring population health during heat events, which has been established in other EU countries for several years [21,22].

Objective 8 (O8): we will further develop and evaluate short-term prediction models of ED usage under varying weather constellations to support operational preparedness and public health decision-making.

Across objectives O1-O5, we will examine differences across vulnerable patient groups and assess regional and socioeconomic disparities using area-level indicators and urban-rural context as potential effect modifiers. A predefined Stuttgart case study will be used for selected secondary analyses that benefit from extended longitudinal coverage and additional pathway detail (eg, basin-valley contrasts).

## Methods

### Study Design and Setting

KlimaNot is a multicenter, registry-based observational cohort study that links routinely collected ED data with hospital-level environmental exposures and comprises both retrospective and prospective components. The retrospective component includes multicenter analyses of ED usage and morbidity as well as the development of syndromic surveillance indicators and prediction models based on linked routine data from participating EDs across Germany. To characterize the structural context of the participating EDs and to assess potential selection effects, key hospital- and location-related characteristics were extracted, including hospital type [23,24], level of emergency care, municipality population size, urban or rural location [25], and total hospital bed size [26] (Table 1).

**Table 1:** Characteristics of participating AKTIN^a^ – EDs^b^ (N=56).

Characteristics		EDs, n (%)
**Hospital type**		
	Community or other hospital	34 (60.7)
	University hospital	22 (39.3)
**Hospital bed capacity**		
	<100	0 (0)
	100-199	3 (5.4)
	200-499	9 (16.1)
	≥500	44 (78.6)
**Emergency care level**		
	Basic	7 (12.5)
	Specialized	9 (16.1)
	Maximum	40 (71.4)
**City population**		
	<100,000	17 (30.4)
	100,000-250,000	17 (30.4)
	250,000-500,000	8 (14.3)
	>500,000	14 (25)
**Location type^a^**		
	Urban	41 (73.2)
	Rural	15 (26.8)

^a^AKTIN: German National Emergency Department Data Registry.

^b^ED: emergency department.

In addition, an ancillary prospective substudy in geriatric clinics collects patient-reported and functional outcomes in the oldest-old population to complement routine registry data. The retrospective and ancillary prospective components are registered separately in German Clinical Trials Registers (DRKS). This protocol prespecifies the main endpoints and analytical framework for the overall study program, while detailed procedures and additional endpoints of the ancillary prospective substudy are specified in its corresponding DRKS record and will be reported separately.

### Data Sources

#### Overview

The study will draw on 4 data sources: routinely collected ED data from the AKTIN ED Registry, additional information from a regional dataset of routine data drawn from the ED Klinikum Stuttgart, survey data from geriatric patients, and environmental exposure data provided by the German Meteorological Service (*Deutscher Wetterdienst* [DWD]) and the Federal Environment Agency (*Umweltbundesamt* [UBA]).

#### Routine ED Records

Retrospective data will be sourced from the AKTIN Registry [27]. The registry is implemented on the underlying AKTIN infrastructure, a nationwide federated data network operated within the Network University Medicine [28]. For the study, a total of 56 EDs throughout Germany, including clinics of different care levels in cities with different population sizes, provide electronic health records from all ED visits documented in the respective ED in the years 2019 to 2024.

Across participating EDs, approximately 6.35 million ED visits will be available for the study period, with an anticipated exclusion of up to 10% due to missing key variables. This retrospective ED dataset contains patient-specific information on age, sex (male, female, or other), and the zip code of the patient’s place of residence, as well as administrative data on mode of transport to the ED, time of admission, and mode of disposition (discharge to home, transfer to other hospital, inpatient admission, or death). Furthermore, it contains the following treatment information: ED diagnoses in the form of *ICD-10-GM* (*International Classification of Diseases and Related Health Problems, Tenth Revision, German Modification*) codes with a labeling of the main ED diagnosis, additional information on diagnosis certainty (secured, suspicion of, condition after or exclusion of diagnose), chief complaint in the form of Canadian Emergency Department Information System – Presenting Chief Complaints (CEDIS-PCL) codes, urgency level coded as Manchester Triage System (MTS) or Emergency Severity Index (ESI), vital parameters, Glasgow Coma Scale (GCS) and isolation status [29-31]. In case of an inpatient admission following the ED visit, inpatient treatment information according to the German social laws regulating the billing of inpatient services (§ 21 Krankenhausentgeltgesetz, KHentG) is available. This information contains the inpatient discharge diagnosis, also coded as *ICD-10-GM*. Multiple ED visits by the same person during the study period cannot be attributed due to data protection requirements.

All ED data will be sourced via established AKTIN registry processes in a standardized format and provided to the study team as a consolidated study dataset for analysis [28]. Before any analyses are conducted, we will perform systematic data quality checks, including verification of variable formats (dates, times, and *ICD-10-GM* codes), plausibility checks for key variables (eg, age range, sex, and time stamps), and identification of potential duplicate records based on site, admission date and time, and local encounter identifiers. Obvious duplicate entries and technically invalid records will be removed in accordance with predefined rules.

#### Stuttgart Regional Dataset

For the analysis of basin-valley differences in Stuttgart, additional regional information beyond the regular dataset is available for the period from 2011 to 2023. This dataset comprises approximately 409,000 cases and enables longer-term analyses. Compared to the AKTIN ED Registry, the dataset provides more detailed information on emergency patients and their treatment. In addition to diagnoses and symptoms, treatment times, duration, diagnostics, place of residence, and physician requirements are recorded. This allows for an analysis of not only the emergency presentation but also the diagnostic and therapeutic effort involved.

#### Prospective Geriatric Data Collection

Additional prospective data on the individual functional level of old patients will be collected at 3 participating study centers (Department of Geriatric Medicine Aachen, Hospital Fürth, and University Clinic for Geriatric Medicine Oldenburg). All geriatric patients admitted to the geriatric ward via the participating EDs at the 3 study centers will be screened and invited to participate in the prospective substudy. Written informed consent will be obtained during inpatient treatment by trained study personnel at the participating centers. In addition to routinely performed comprehensive geriatric assessment, the substudy will collect standardized patient-reported and functional outcomes, including the Parker Mobility Score (mobility) [32], the SF-12 Health Survey (health-related quality of life) [33], and parts of the questionnaire on social situation [34]. A project-developed questionnaire will assess self-reported heat exposure and heat-related problems in the days preceding hospital admission. A follow-up survey approximately 6 weeks after discharge will assess longer-term outcomes, such as reduced mobility and increased level of dependency (eg, discharge to a nursing home). In addition, inpatient data will be used to describe specific characteristics of the geriatric cohort using the Hospital Frailty Risk Score [35] and investigate outcomes as length of stay and in-hospital mortality. To maximize recruitment and follow-up adherence in this vulnerable cohort, only geriatric centers with established research experience are included. Study personnel will be dedicated to the substudy, experienced in geriatric care, and located directly on the wards to enable repeated opportunities for study information, support with questionnaires, and communication with relatives or legal representatives when required. Contact information for follow-up will be updated by the day of discharge, particularly in case of changes in the discharge destination (eg, nursing home). Follow-up will be conducted within a 2-week contact window around the 6-week time point, with repeated contact attempts if needed. Based on treatment capacities and prior patient volumes at the participating centers, we estimate that approximately 281 patients will meet the inclusion criteria during the planned recruitment period. Based on experience from previous geriatric studies, we expect that approximately 50% will consent to participate (approximately 139 participants). For the 6-week follow-up, a dropout rate of approximately 50% is anticipated.

#### Environmental Data

The retrospective weather and environmental data are collected from freely accessible datasets provided by the German Meteorological Service (DWD) and the Federal Environment Agency (UBA). The DWD provides data sets comprising hourly and daily aggregated data on air temperature, humidity, wind speed, and precipitation. The Federal Environment Agency provides datasets on air quality at an hourly level on ozone (O₃), nitrogen dioxide (NO₂), particulate matter (PM10 [particulate matter with a diameter ≤10 µm] and PM2.5 [particulate matter with a diameter ≤2.5 µm]), and sulfur dioxide (SO_2_). For the regular use case Stuttgart, data on global radiation, radiation balance, as well as UV-A and UV-B radiation will also be available. The weather and environmental data of Stuttgart will be sourced from an environmental monitoring station located in the valley basin, approximately 1.3 km from the clinic. In addition, pollen count data will be acquired from the German Pollen Information Service foundation (*Polleninformationsdienst* [PID]). The PID operates various pollen measuring stations in Germany, where the pollen concentration of various allergenic plants and trees is measured and counted on a daily basis. The pollen types with the highest allergic potential for humans will be selected together with experts from the PID.

#### Area-Level Socioeconomic Deprivation

Area-level socioeconomic deprivation will be operationalized using the German Index of Socioeconomic Deprivation (GISD), a composite measure developed by the Robert Koch Institute that summarizes relative regional deprivation across education, employment, and income dimensions and is available at multiple spatial levels (eg, municipality and district) for different years. Patients’ residential postal codes will be mapped to GISD values and used to assess socioeconomic gradients and potential effect modification of heat–outcome associations.

### Outcomes

We defined one primary objective with a corresponding primary outcome (O1), a set of secondary clinical and usage objectives and outcomes (O2-O5), and exploratory methodological objectives and outcomes related to a prospective substudy, syndromic surveillance, and prediction modeling (O6-O8). All exposure definitions and analytical decisions are specified in the statistical analysis plan.

### Primary Outcome: Daily All-Cause ED Usage (O1)

We will quantify the number of ED encounters per participating hospital and calendar day (hospital-day level; AKTIN encounter identifier aggregated by clinic × date).

### Secondary Outcomes (O2-O5)

#### Daily Admission Rate From the ED (O2)

We will use the proportion of ED encounters resulting in inpatient admission per hospital and day (hospital-day level; derived from encounter disposition and admission fields using AKTIN encounter identifier aggregated by clinic×date).

#### Syndromic and Diagnostic Case-mix (O3)

We will quantify daily counts and proportions of prespecified diagnostic and syndromic categories, including heat-sensitive conditions and shifts in major diagnosis group distributions (hospital-day level; derived from ED *ICD-10-GM*, inpatient discharge *ICD-10-GM*, and CEDIS-PCL).

#### Access Pathways (O4)

We will analyze the daily distribution of referral sources and mode of transport to the ED (hospital-day level; derived from encounter-level pathway variables such as transport and referral).

#### Severity Proxies (O5)

We will use daily distribution of acuity and clinical severity indicators at presentation (hospital-day level; derived from triage categories, high-acuity proportions, and additional severity proxies such as vital signs or Glasgow Coma Scale).

#### Ancillary Prospective Geriatric Substudy: Heat-Related Symptom Burden in Older Adults (O6)

We will characterize patient-reported heat-related symptoms and functional impact using structured questionnaires at prespecified assessment time points (participant-level, derived from questionnaires on quality of life, mobility, social background, and heat-related behavior).

#### Exploratory Outcomes (O7-O8)

##### Syndromic Surveillance (O7)

We will create syndrome definitions to identify cases of climate change-related ED visits in the routine data and validate their performance by comparing them with different reference data sources (heat-related syndromic indicators).

##### Prediction Model Performance (O8)

We will quantify accuracy of weather-informed models forecasting daily ED usage and related usage metrics (hospital-day forecast level; evaluated across hospitals, dates, and times).

Across objectives O1-O5, we will evaluate whether associations differ across vulnerable patient groups (eg, age, sex, and multimorbidity proxies) and by area-level vulnerability indicators, including socioeconomic deprivation (GISD) and urban–rural context. All exposure definitions and analytical decisions are specified in the statistical analysis plan.

### Data Linkage

**Figure 1:**
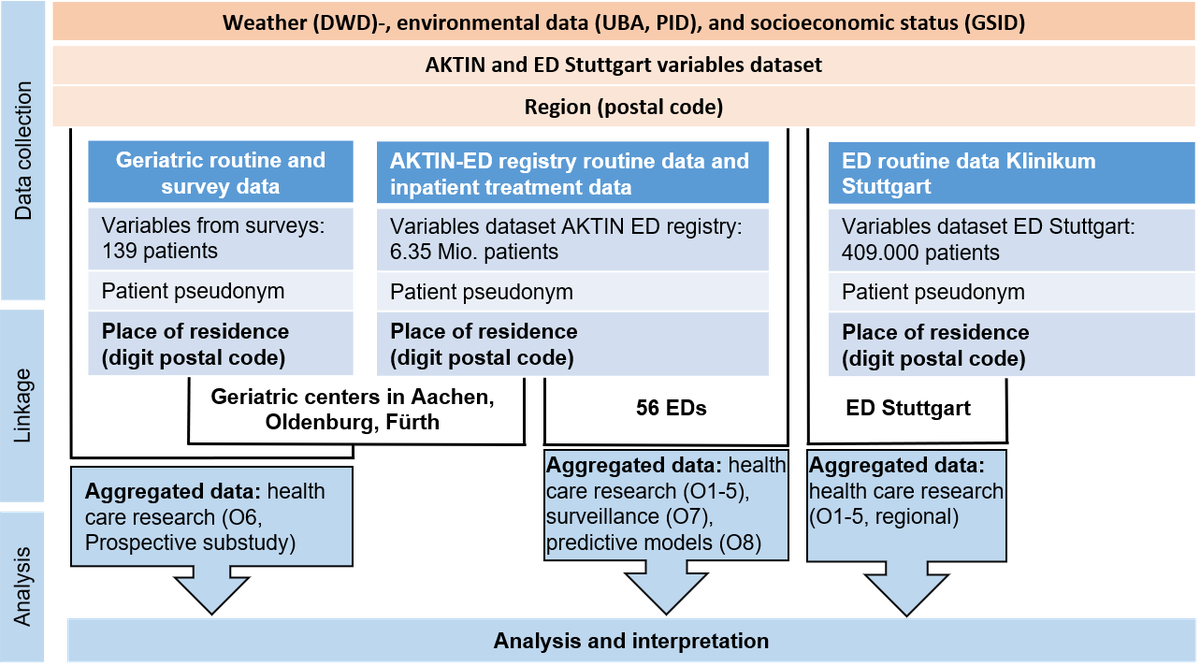
Overview of data sources, linkage, and analysis. DWD: German Meteorological Service (Deutscher Wetterdienst); ED: emergency department; GSID: German index of socioeconomic deprivation; PID: Polleninformationsdienst; UBA: Federal Environment Agency (Umweltbundesamt).

The linkage between patient data and environmental data is established at the hospital level. For each hospital, the nearest weather station with the smallest air distance is identified, ensuring that there are fewer than 500 missing values within the time interval of 2019-2024. Due to the limited spatial availability and completeness of air quality data, air data are matched solely based on proximity to the hospital, accepting some missing values. Both the weather and air quality data are provided as hourly records, as well as in a summarized format with daily minimum, maximum, and mean values. The spatial granularity is selected to be as fine as possible. However, a finer granularity is not feasible due to data policy restrictions on patient data and the limitations of the environmental measurement grid. Under the assumption that environmental phenomena generally occur on a large scale and that most patients choose the nearest ED in case of an emergency, the linked data provide at least an approximate overview of the environmental influences to which patients were exposed before the onset of their illness. Following scientific best practices and ensuring reproducibility, the Python code used for the data linkage is publicly available on GitHub [35]. The pollen stations will be selected in consultation with experts from the PID, considering data completeness as well as proximity to the ED (Figure 1).

### Data Protection

A scientific advisory board has been established, comprising representatives from medical associations (including the German Interdisciplinary Association for Intensive Care and Emergency Medicine and the German Society for Interdisciplinary Emergency and Acute Medicine), clinical experts specializing in emergency medicine, and a patient representative from the German Coalition for Patient Safety (*Aktionsbündnis Patientensicherheit* e.V., Berlin). Additional experts from the Federal Office for Citizen Protection and Disaster Support (*Bundesamt für Bevölkerungsschutz und Katastrophenhilfe*) and the DWD are involved. The purpose of this board is to offer scientific guidance for the project, advocate for the interests of patients, and assist in implementing the outcomes.

### Ethical Considerations

#### Ethics Approval

This study was approved by the leading ethics committee at the Medical Faculty of the University of Magdeburg (29/24 dated March 08, 2024), as well as by the institutional ethics committee at RWTH Aachen (24-333 dated October 10, 2024).

#### Informed Consent

The use of the ED data has been approved by the AKTIN Data Use and Access Committee and is subject to AKTIN’s data protection regulations. Written informed consent will be obtained from all patients participating in the ancillary prospective geriatric substudy. Both the retrospective registry-based study and the ancillary prospective geriatric substudy are registered in the DRKS and are governed by their respective approvals (AKTIN data access for retrospective analyses; site-specific ethics and informed consent for the prospective substudy).

#### Privacy and Confidentiality

Data are processed in compliance with applicable data protection regulations. The dataset used for analysis is pseudonymized at source and direct identifiers are not available to the study team. Data transfer and storage are secured (encrypted transfer, access restricted to authorized personnel, analysis on secure servers), and results are reported only in aggregated form.

#### Compensation

Geriatric participants will receive no compensation.

### Statistical Methods

The statistical analysis will be conducted using R (R Development Core Team).

#### Descriptive Overall Population Analyses

The descriptive analysis of the overall population will characterize the overall cohort and support the planning of relevant subgroup analyses. It will include key aspects of the cohort based on multiple variables, including sociodemographic factors (age and gender), presenting complaints (CEDIS-PCL), initial triage assessment (MTS or ESI), patient referral pathways, hospital stay duration, discharge outcomes, diagnostic categories (*ICD-10-GM*), and socioeconomic status (GISD). Continuous variables will be summarized using mean (SDs) or median (IQRs), and categorical variables as counts and percentages. Distributions will be presented graphically (eg, histograms and density plots).

#### Statistical Modeling of Environmental Factors on ED Visits (O1-5)

The number of all-cause ED visits in relation to temperature and environmental factors will be investigated using a 2-stage analysis approach. In the first stage, given the available data, we plan to fit the site-specific number of daily ED visits using a quasi-Poisson regression model including a distributed lag nonlinear model for daily temperature to capture delayed temperature effects, using appropriate splines and lags [34].

These models aim to adjust for seasonal trends in temperature and air quality using smooth functions and will additionally include day of week, public holidays, and further covariates (O₃, NO₂, particulate matter [PM10 and PM2.5], SO_2_, UV-A, UV-B radiation, and predefined pollen). Heat days, one of the main focuses of this study, will be defined a priori based on clinic-specific temperature distributions (days with mean air temperature above the 95th percentile), and alternative operationalizations (eg, heat index, tropical nights, and heatwaves) will be examined in prespecified sensitivity analyses. The analysis is planned to be stratified by age group (0-5 years, 6-11 years, 12-17 years, and ≥65 years). In the second stage, ED-specific distributed lag nonlinear model coefficient vectors are planned to be pooled using a random-effects meta-analysis approach to obtain an overall heat exposure–response association and to quantify between-site heterogeneity [34]. As sensitivity analysis, we will assess the robustness of our modeling choices, including alternative lag windows, splines, and alternative number of knots for temperature and lag. We will also investigate the sensitivity when using other alternative stratification strategies and missing data handling. A detailed statistical analysis plan will be prepared in advance of the data analysis.

#### Ancillary Prospective Geriatric Substudy (O6)

Analyses of the prospective geriatric substudy will be conducted separately from the retrospective registry-based analyses and follow prespecified endpoints and analytical procedures as defined in the substudy protocol and DRKS registration. We will summarize baseline characteristics and patient-reported and functional outcomes using appropriate descriptive statistics. Associations between heat exposure measures and substudy outcomes will be evaluated using regression models suited to the outcome scale (eg, linear, logistic, or ordinal models, as applicable), with effect estimates reported alongside 95% CIs. Models will adjust for key prespecified covariates available in the substudy dataset (eg, age, sex, and frailty-related measures), and sensitivity analyses will address missing data and loss to follow-up where appropriate.

#### Surveillance (O7)

To create the syndrome definition, a literature search on ED visits during heat events will be conducted to identify relevant *ICD-10-GM* and CEDIS-PCL codes. The feasibility and validity of the created syndrome definitions will then be assessed using the above-described retrospective ED data linked with environmental data. We will perform descriptive analysis of the identified cases of the health indicator of interest (eg, heat-related ED visits) using time series and exposure-outcome plots to visualize a potential association between the frequency of codes and temperature. Logistic regression analyses will be used to quantify these associations and account for uncertainty. The results will be discussed with clinical experts, and the syndrome definition will be adjusted in an iterative process until one indicator is finalized. To validate the indicator, time series of the syndrome definition will be compared with temperature time series, and mixed model logistic regression analyses will be conducted again to quantify the associations and uncertainty between trends of identified cases and changes in temperature. Furthermore, we will be looking into other external data sources, such as heat mortality and heat warnings from the DWD, to assess if we can perform additional validation analysis by comparing them with the heat indicator created in this study.

#### Prediction Model (O8)

The objective is to develop mathematical models using machine learning methods to enable simulation-based analyses, such as weather-dependent predictions. These models will vary in granularity and capture correlations between environmental data and disease-specific demographic factors, such as age and sex. Feature selection will be tailored to different disease types, incorporating demographic characteristics and clinical patient information. Based on this analysis, customized data-driven machine learning models will be developed. Various model types, including support vector machines, feedforward neural networks, and recurrent neural networks, will be explored. The proposed architectures were selected based on their strengths for the prediction task at hand: support vector machines provide robust performance in high-dimensional feature spaces with strong regularization properties; Feedforward neural networks offer flexibility in modeling complex nonlinear relationships between environmental and demographic variables; and recurrent neural networks are specifically suited for capturing temporal dependencies and lagged effects inherent in time series data such as seasonal ED usage patterns. These models will be trained and cross-validated using supervised learning on ED data. A literature review will be conducted to identify models that integrate expert knowledge, with a particular focus on time series models incorporating climate data for forecasting ED usage. An initial approach may involve time series regression with climate variables, allowing for the prediction of seasonal trends. Additionally, daily aggregated environmental data (see section *Data Linkage*) will be incorporated into the modeling process. This dual approach mitigates project risks by combining machine learning as a black-box model with an interpretable time series approach. The integration of both methodologies will enhance insights, particularly in feature selection. To ensure reproducibility, all source code will be made publicly available via the project’s GitHub repository [35].

## Results

The retrospective analysis of the overall study population has already been completed. However, results will be published separately and are not reported here. The health care research analysis will be finalized by April 1, 2026. The analysis of data from the multicenter prospective study is scheduled for completion by October 31, 2026. The evaluation of the usability of AKTIN data for near real-time public health surveillance in the context of extreme weather events and the implementation of a prototype module for localized application of predictive models will both be finalized by June 30, 2026.

## Discussion

### Anticipated Findings

This multicenter, registry-based observational study will provide valuable insights into the impact of climate change-related weather phenomena on emergency and acute care services in Germany. Because ED contacts capture acute decompensations and time-critical presentations at the front door of the health system, routinely collected ED data can provide early signals of weather-sensitive health impacts and short-term burden. These impacts often become evident before they are fully reflected in downstream outcomes such as hospital discharge diagnoses or mortality statistics. Within this sentinel framing, we anticipate that extreme heat will be associated not only with changes in overall ED volume, but also with measurable shifts in the composition of presentations and care pathways. We assume that heat exposure will be associated with measurable increases in overall ED usage and diagnosis-specific patterns consistent with heat sensitivity (eg, dehydration and heat illness, renal diseases, and cardiopulmonary diseases). We further expect that associations will be heterogeneous across patient groups, with stronger relative effects in vulnerable populations such as children and adolescents, and older patients or those with multiple comorbidities, consistent with prior evidence on heat-related morbidity gradients [17,36,37]. Prior research has documented associations between high temperatures and a broad range of acute morbidity outcomes, including ED visits and emergency hospitalizations, with differential impacts by age and comorbidity [15,21,38,39]. However, many large-scale studies still rely on mortality or hospital admission endpoints, and ED-based analyses often focus on visit counts or diagnosis groups, which can limit the characterization of operationally relevant care pathways and disposition and admission patterns as distinct outcomes [21,38]. In particular, multicenter ED studies that jointly assess volume, case mix, and pathway-related endpoints remain limited. ED-based syndromic surveillance can support situational awareness since EDs capture acute presentations in near real time. In Germany, routine data from the AKTIN ED registry provide an important basis for such monitoring [27]. In this project, syndromic surveillance will be operationalized using *ICD-10-GM* and CEDIS-PCL codes, and the proposed heat-related syndrome definition will be developed and empirically tested. Syndromic surveillance of heat-related visits can complement predictive models by providing timely signals for estimating and tracking heat-related illness at the population level [22]. The main goal of this study is to produce results that can be compared across different sites and that are useful for preparedness and prevention. The findings may help EDs plan their staffing and capacity during forecasted heat days and heat periods, supporting public health surveillance and guiding targeted prevention for high-risk groups. By looking not only at ED usage, but also at changes in case mix and pathways of care, the project aims to build a more complete preparedness approach that reflects how acute care works across sectors such as ED, emergency medical service, outpatient care, and inpatient beds. The results are likely to apply best to ED settings that are similar to the participating sites in terms of documentation practices, catchment area, and the quality of linking ED data to environmental exposure data.

### Strength and Limitations

Our study addresses an urgent global challenge by focusing on health risks associated with climate change and provides much-needed evidence on the effects of weather-related phenomena on ED usage in Germany. By using routine data, the study reflects real-life clinical conditions and makes efficient use of existing resources. The integration of both retrospective and prospectively collected data allows for a more comprehensive understanding of acute care demands, particularly among vulnerable groups. A major strength of this project is the use of standardized, routinely collected ED registry data that can support multicenter analyses across heterogeneous sites [28]. However, there are also limitations, as the AKTIN registry does not fully represent the entire German hospital landscape, which may limit the generalizability of the findings. Compared with the German hospital landscape, the participating EDs include a substantially higher proportion of university hospitals. In addition, the sample comprises a high share of large hospitals and is predominantly urban.

Moreover, the expected sample size for the prospective geriatric survey is not powered to detect statistically significant effects but is designed to offer exploratory insights into the acute care needs of older adults.

### Dissemination

Findings of the study will be made available as oral or poster presentations at scientific conferences, through publications in peer-reviewed journals, and will be shared with relevant clinical, public health, and policy stakeholders. In addition, resource and care relevant results related to ED overcrowding, patient referral, and morbidity that are derived from the health care research analyses, including validated syndromic surveillance, and from prediction modeling, will be presented to participating EDs through an interactive web application. Using the application, the EDs should be able to estimate what usage can be expected under the assumption of certain weather constellations.

## Data Availability

The ED data are managed by the German Emergency Department Data Registry (AKTIN, [40]) and are subject to data protection regulations. As such, they are not publicly available but can be requested directly from the registry under applicable conditions. Weather data are publicly accessible via the DWD website. All code used for data processing and analysis is available in our GitHub repository [35].

## Abbreviations

CEDIS-PCL: Canadian Emergency Department Information System – Presenting Complaint List
DRKS: German Clinical Trials Register
DWD: German Meteorological Service (Deutscher Wetterdienst)
ED: emergency department
ESI: Emergency Severity Index
GCS: Glasgow Coma Scale
GISD: German Index of Socioeconomic Deprivation
ICD-10-GM: International Statistical Classification of Diseases and Related Health Problems, Tenth Revision – German Modification
MTS: Manchester Triage System
PID: German Pollen Information Service foundation (Polleninformationsdienst)
PM10: particulate matter with a diameter ≤10 µm
PM2.5: particulate matter with a diameter ≤2.5 µm
UBA: Federal Environment Agency (Umweltbundesamt)
